# Multimodal Spatial Transcriptomics Reveals the Developing Human Liver Niche at Single-Cell Resolution

**DOI:** 10.1016/j.gastha.2026.100893

**Published:** 2026-02-03

**Authors:** Michael Bailey, Michael Leon, Isabella Rosso, Carolyn Sangokoya

**Affiliations:** 1 Customer Experience Lab, Bruker Spatial Biology, Seattle, Washington; 2 Neurogenetics Research Unit, Max Planck Institute, Frankfurt, Germany; 3 Department of Pathology, University of California San Francisco, San Francisco, California; 4 Eli and Edythe Broad Center of Regeneration Medicine and Stem Cell Research, Center for Reproductive Sciences, University of California San Francisco, San Francisco, California; 5 Liver Center, University of California San Francisco, San Francisco, California

**Keywords:** CXCL12, Human Liver Development, Spatial Biology, Spatial Transcriptomics, SPP1

## Abstract

**Background and Aims:**

A comprehensive understanding of the liver niche during development provides insights into a unique, naturally occurring multifunctional multicellular environment. We applied spatial transcriptomic and histologic approaches to identify and histologically validate RNA-based markers in human liver tissue during a critical developmental window when the liver serves as the primary site of hematopoiesis, just prior to the rapid growth phase of the third trimester. During this period, the developmental liver niche uniquely supports the coexistence of endodermal- and mesenchymal-derived cell populations that coordinate hematopoietic development and hepatocyte function.

**Methods:**

Nine formalin-fixed paraffin-embedded human developmental liver samples underwent histologic processing, staining, and evaluation prior to single-cell resolution spatial molecular imaging. Spatial transcriptomic profiling was followed by single-cell transcriptomic analysis and validation using multiplexed RNA fluorescent single-molecule in situ hybridization.

**Results:**

Single-cell spatial imaging identified epithelial, hematopoietic, endothelial, and stromal cell populations within shared microenvironments. This approach enabled molecular capture and histologic confirmation of 2 complex developmental phenomena: gene expression associated with the developing ductal plate and CXC motif chemokine ligand localization to the hepatic hematopoietic stem cell niche.

**Conclusion:**

Integration of markers identified through spatial transcriptomics will expand molecular panel design and enable more precise characterization of developmental biliary biology and pathobiology. Importantly, RNA-based understanding of the functional diversity and in situ spatial organization of the developmental liver niche will also be essential for authenticating and re-engineering these complex regenerative environments in vitro.

## Graphical abstract

**Figure:**
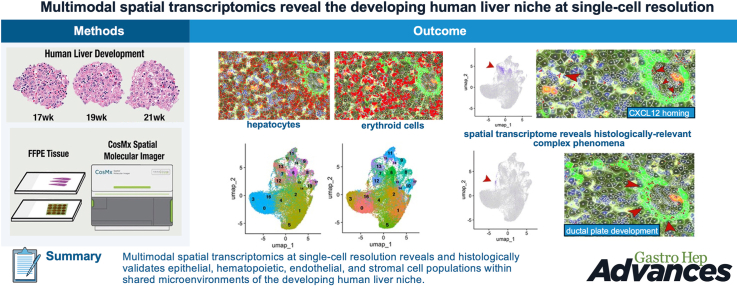

## Introduction

In the adult, the liver is the largest gland in the body with both exocrine (bile production) and endocrine (hormone secretion) functions. The adult liver also performs drug detoxification, metabolic control, glycogen storage, urea metabolism, and secretion of plasma proteins. However, in development, the liver also has very different functions as (1) the vascular connection between placental vessels and the heart and (2) the site of hematopoiesis (first detected around the fifth week of gestation). This environment supports the expansion and differentiation of hematopoietic stem and progenitor cells.^1^, ^2^, ^3^ Liver hematopoietic progenitors are essential to support the developing human until the end of gestation, with the bone marrow taking over after birth. Developmental liver hematopoiesis undergoes a similar differentiation process as marrow progenitors after birth but with distinct differences in response to cytokines and proliferative capacity.^4^, ^5^, ^6^, ^7^

In the developing human liver, hepatocytes develop in an environment composed of many cell types. By the second trimester, the cell types that build these complex microenvironments include epithelial cells (hepatocytes and biliary progenitors), a hematopoietic niche including myelolymphoid progenitors, erythroid progenitors, resident macrophages, and stromal cell populations of mesenchymal origin including stellate cells, fibroblasts, myofibroblasts, vascular smooth muscle, and endothelial cells. This unique microenvironment—with cells of endoderm origin, mesenchymal origin, and the hematopoietic niche—nurtures the successful maturation of these cell types and could model features helpful to induce and validate successful induction of these cells in vitro and ex vivo.

In recent years, in-depth single-cell transcriptomics studies have led to a more comprehensive understanding of the landscape of the developing human liver.^8^, ^9^, ^10^, ^11^, ^12^ While dissociation of cells before sequencing results in the loss of the in situ cell organization within the tissue, the advent of in situ spatial transcriptomics at single-cell resolution with high-resolution imaging^13^ integrates spatial, single-cell, and imaging capabilities and allows for an additional revealing of the molecular underpinnings of complex phenomena at the tissue level.

## Materials and Methods

### Tissue Samples, Histology, and Microscopic Analysis

Basic histology and hematoxylin/eosin staining of tissue from 9 formalin-fixed paraffin-embedded (FFPE) human liver tissue samples ranging from 17 to 21 weeks’ gestational age was performed by histology staff of the UCSF Department of Pathology. Initial evaluation of the sections was performed by a board-certified and expert liver pathologist (C.S.) prior to use in studies.

### Single-Cell Spatial Transcriptomics and Cell Module Enrichment

Spatial transcriptomic profiling of FFPE liver sections was performed using the CosMx Spatial Molecular Imager, as previously described.^13^ The 1000-plex CosMx Human Universal Cell Characterization RNA Panel was used to measure transcript expression, 4′,6-diamidino-2-phenylindole, and immunofluorescence staining for cluster of differentiation 45 (CD45), pan-cytokeratin (PanCK), and cluster of differentiation 3 (CD3). The BioTuring Lens Cloud Platform was used to visualize and perform preliminary quality control analyses of segmentation, staining, and image clarity. Supervised initial cell typing was performed using relevant liver single-cell sequencing studies and confirmatory histologic analysis by a trained pathologist (C.S.). The following were included as additional variables used to subcluster cells: mean PanCK staining intensity, mean CD45 staining intensity, and mean CD3 staining intensity. Following quality control and initial cell typing, all single cells were exported and analyzed by the Seurat package (v5.1.0)^14^ in the R environment (v4.4.1)^15^ using the FindNeighbours, FindClusters, and FindMarkers functions to independently identify clusters and marker genes for each cluster based on single-cell analysis of the imaging data. Cell module enrichment was calculated using the AUCell algorithm to identify gene modules in the single-cell data.^16^

### RNA In Situ Hybridization and Image Acquisition

Multiplexed fluorescent single-molecule in situ hybridization (smFISH) was performed on tissue from the same paraffin blocks used for CosMx spatial molecular imaging analysis using the RNAscope Multiplex v2 assay kit (ACDBio) as per the manufacturer’s instructions. Hybridization was performed with probes against *Hs*-*SPP1* (420101; ACDBio) and *Hs*-*CXCL12* (422991; ACDBio). Images were visualized and captured using an Echo Revolve Fluorescence Microscope.

## Results

### Multimodal Spatial Molecular Imaging at Single-Cell Resolution

**Figure 1:**
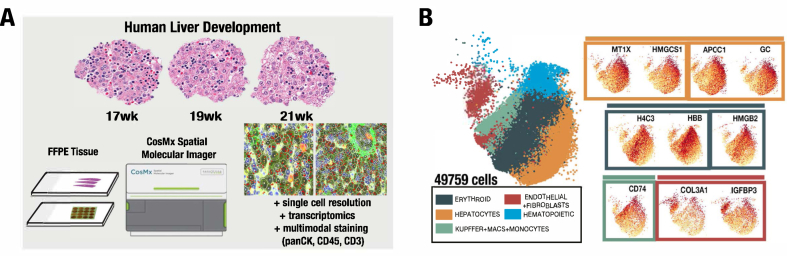
Single-cell spatial transcriptomic profiling reveals diverse cell types of developmental liver cell niche. (A) Schematic showing range of sample gestational ages and procedural flow from formalin-fixed paraffin-embedded (FFPE) tissue (left) to processing by the spatial molecular imager (middle) and analysis of images (right). (B) A total of 49,759 cells from 9 samples resolved by the spatial imager and analyzed. A UMAP projection of these cells is shown and is color coded to represent hepatocytes, hematopoietic and erythroid cells, endothelial cells and fibroblasts, Kupffer cells, macrophages, and monocytes. Representative markers of each of these populations are highlighted (right). UMAP, uniform manifold approximation and projection.

**Figure 2:**
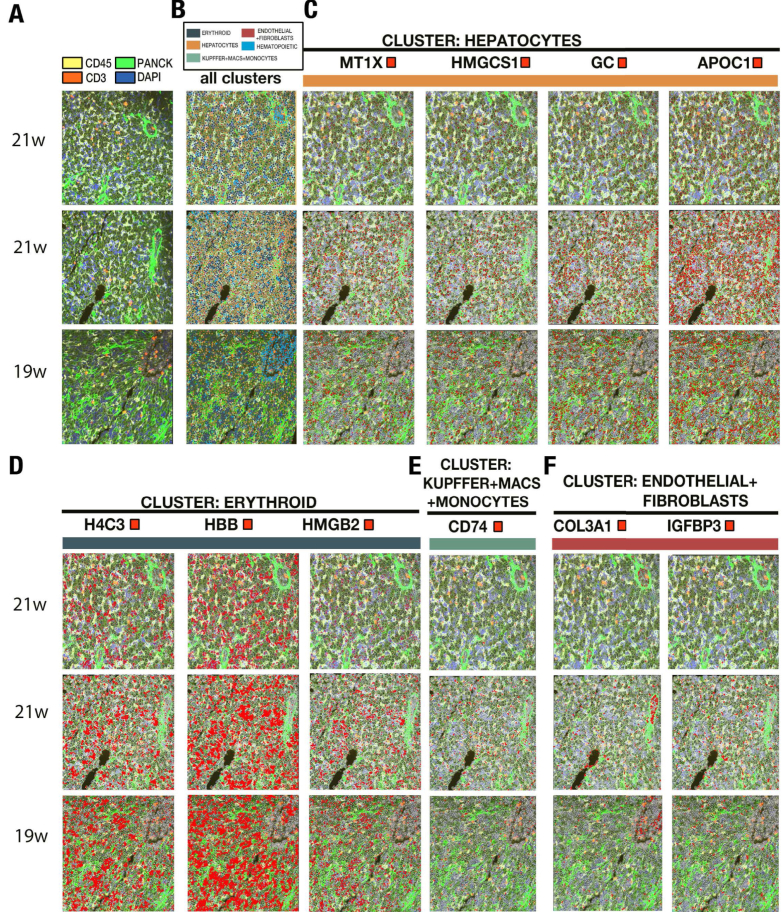
Multimodal spatial imaging identifies cell type cluster expression at single-cell resolution with histologic confirmation. Images depicting (A) first column, multimodal staining with CD45, Pan-CK, CD3, and DAPI, from 3 individual samples. (B) Second column, preliminary cell typing clusters color coded to represent hepatocytes, hematopoietic and erythroid cells, endothelial cells and fibroblasts cells, Kupffer cells, macrophages, and monocytes, from 3 individual samples. (C) Third through sixth columns, representative markers for hepatocytes (*MT1X*, *HMGCS1*, *GC*, *APOC1*, respectively), with transcripts in red at single-cell resolution, from 3 individual samples. (D) First through third columns, representative markers for erythroid cells (*H4C3*, *HBB*, *HMGB2*, respectively), with transcripts in red at single-cell resolution, from 3 individual samples. (E) Fourth column, representative markers for Kupffer cells, macrophages, and monocytes (*CD74*), with transcripts in red at single-cell resolution, from 3 individual samples. (F) Fifth and sixth column, representative markers for endothelial cells and fibroblasts (*COL3A1*, *IGFBP3*) with transcripts in red at single-cell resolution; from 3 individual samples. *APOC1*, apolipoprotein C1; *CD74*, cluster of differentiation 74; DAPI, 4′,6-diamidino-2-phenylindole; *GC*, GC vitamin D binding protein; *HBB*, hemoglobin subunit beta; *H4C3*, H4 clustered histone 3; *HMGB2*, high mobility group box 2; *HMGCS1*, 3-hydroxy-3-methylglutaryl-CoA synthase 1; *IGFBP3*, insulin-like growth factor binding protein 3; *MT1X*, metallothionein 1X.

Samples of FFPE human liver tissue ranging from 17 to 21 weeks’ gestational age were sectioned for histologic and spatial molecular analysis. A total of 49,759 cells over 9 tissue microarray cores were identified after processing by the CosMx Spatial Molecular Imager,^13^ a spatial transcriptomic imaging platform that processes, stains, washes, images, and provides segmented quantitative digital transcriptomic readout of the FFPE tissue at single-cell resolution with multimodal analysis for PanCK, CD45, and CD3 (Figure 1A). Interactive visualization using the BioTuring Lens spatial transcriptomics analysis platform allowed for preliminary analysis by in situ cell typing, histologic confirmation, and transcript expression. Based on this preliminary analysis informed by previous human single-cell RNA sequencing studies,^8^^,^^17^, ^18^, ^19^ 5 major spatially informed transcriptomic clusters were generated, composed of hepatocytes, erythroid cells, endothelial cells, fibroblasts, hematopoietic cells, Kupffer cells, macrophages, and monocytes (Figure 1B). Hematoxylin/eosin staining of the liver tissue highlights the hematopoietic cell niche between cords of hepatocytes at this stage of development (Supplementary Figure 1) and staining for PanCK, CD45, CD3, and 4′,6-diamidino-2-phenylindole without cell typing (Figure 2A) captures the cell and tissue architecture. After labeling the single cells of the tissue by initial cell typing, a closer look at the histologic samples gives histologic confirmation of spatially informed transcriptomic major cell type clusters (Figure 2B, enlarged in Supplementary Figure 2A).

### Diverse Cell Populations Revealed by Transcript-Based Multimodal Analysis With Integrated Histologic Confirmation

Our study samples capture the rich context of the major cell types of the liver. Transcript expression is highlighted in red for selected representative markers in hepatocytes (*MT1X*, *HMGCS1*, *GC*, *APOC1*), erythroid cells (*H4C3*, *HBB*, *HMGB2*), Kupffer/macrophages/monocytes (*CD74*), endothelial cells, and fibroblasts (*COL3A1*, *IGFBP3*) (Figure 2 and Supplementary Figure 2). In these sections, spatial histologic confirmation demonstrates the CD45-positive cells (yellow) representing a significant population of the hematopoietic cell niche as cords of cells around the PanCK-positive cells (dark green). Groups of smaller cells with prominent nuclei typically found within the stem cell niche indeed express prominent hemoglobin transcripts of erythroid progenitors and stain very blue due to the high concentration of small cells with high nuclear-to-cytoplasmic ratios (Figure 2) The vasculature stains bright green with highlighted collagen type I alpha 1 chain expression on the inside surface, an expected and confirmatory finding as this transcript is highly expressed in vascular smooth muscle cells (Figure 2 and Supplementary Figure 2). *CD74* expression, which regulates B- and T-cell development, is captured across CD45-positive cells abutting the hematopoietic stem cell niche (HSC; Supplementary Figure 2F). Here, we leverage histologic spatial information with transcriptomics and multimodal analysis for easily confirmatory preliminary analysis of these cellular environments at single-cell resolution.

### Profiling by Single-Cell RNA Transcriptomic Analysis With Histologic Confirmation by Spatial Imaging

**Figure 3:**
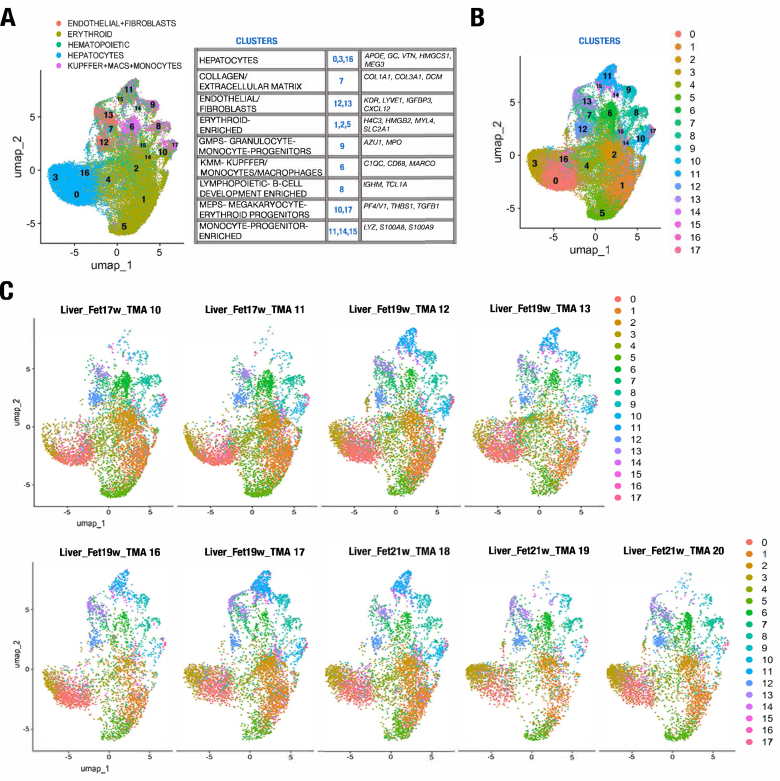
Spatial profiling captures diverse cell types in the developmental liver cell niche at single-cell resolution. (A,B) UMAP projection of the 49,759 cells in this study, demonstrating 17 clusters representing 9 cell types, colored by preliminary cell typing in and after informed Seurat-based clustering. (C) RNA signature UMAP projections generated for each individual sample of the study. UMAP, uniform manifold approximation and projection.

After this preliminary analysis with histologic confirmation, in-depth single-cell transcriptomic profiling was performed de novo independent of original histology-confirmed cell typing using Seurat^14^ to generate uniform manifold approximation and projection projections, identify clusters, and explore expression data to fine-tune these clusters. Here, we were able to expand characterization of the hematopoietic cell niche by utilizing guidance from previous single-cell studies.^8^^,^^17^, ^18^, ^19^ Identified clusters were again informed by previous studies^8^^,^^17^, ^18^, ^19^ that added resolution and further revealed the diverse functional cell types of the developmental liver cell niche captured in this study (Figure 3A and B). RNA signature projections were generated individually from each of the samples of the study, demonstrating similar patterns between these histologically normal samples over the 17 to 21-week developmental time (Figure 3C). Marker genes identified by cluster analysis (Figure 3A and Supplementary Figure 3) highlight markers in hepatocytes (*APOE*, *GC*, *MEG3*, *VTN*, *HMGCS1*), erythroid cells (*H4C3*, *HMGB2*, *MYL4*, *SLC2A1*), Kupffer cells/monocytes/macrophages (*C1QC*, *CD68*, *MARCO*), collagen/extracellular matrix (*COL1A1*, *COL3A1*, *DCN*), lymphopoietic/B-cell development (*IGHM*, *TCL1A*), granulocyte-monocyte progenitors (*AZU1*, *MPO*), megakaryocyte/erythroid progenitors (*PF4/V1*, *THBS1*, *TGFB1*), monocyte progenitors (*LYZ*, *S100A8*, *S100A9*), and endothelial/fibroblasts (*KDR*, *LYVE1*, *IGFBP3*, *CXCL12*).

### Visualizing Complex Phenomena Revealed by Spatial Molecular Imaging

**Figure 4:**
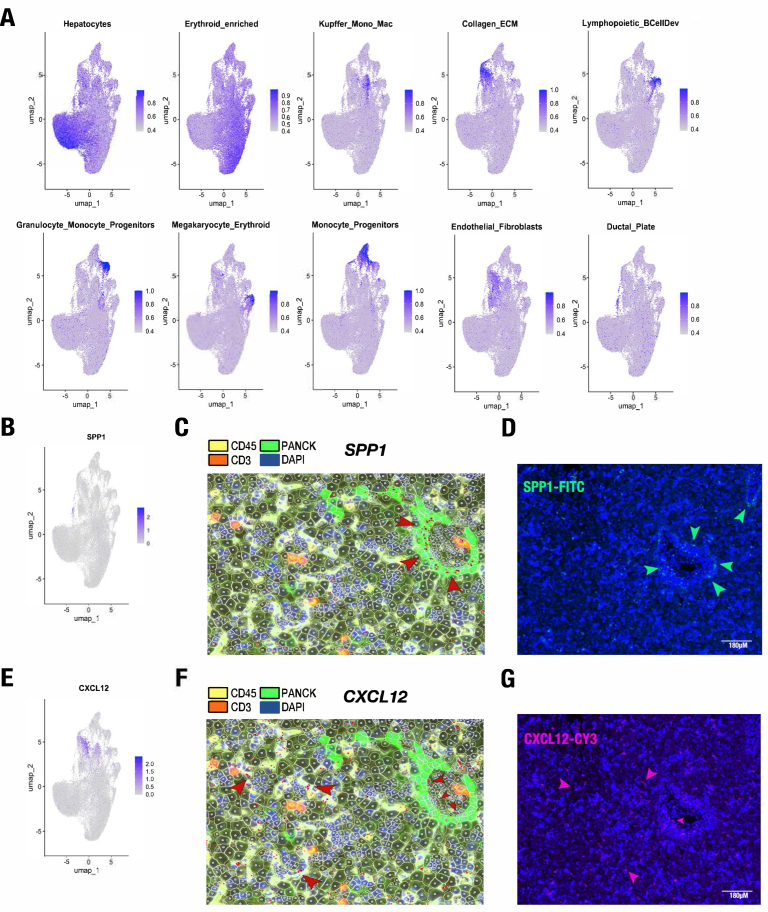
Cell type module enrichment highlights clusters, ductal plate expression, and CXCL12 stem cell niche homing. (A) Cell type module enrichment using multiple genes identified as enriched in specific clusters as shown by UMAP projection. Intensity colored by module enrichment. (B) Ductal plate enrichment identified spatially is enriched for *SPP1* as shown by UMAP projection. Intensity colored by module enrichment. (C) Histologic image with multimodal staining shown and *SPP1* transcripts shown in red at single-cell resolution. (D) RNA single-molecule in situ hybridization analysis of *SPP1* performed on a different section. (E) CXCL12 expression corresponds with endothelial cell-type module enrichment shown in (A), shown here by UMAP projection. Intensity colored by module enrichment. (F) Histologic image with multimodal staining shown and *CXCL12* transcripts shown in red at single-cell resolution. (G) RNA single-molecule in situ hybridization analysis of *CXCL12* performed on a different section. UMAP, uniform manifold approximation and projection.

Cell type module enrichment allowed for analysis of multiple genes as modules (Figure 4A). Using this analysis, we identified markers for 2 complex phenomena by spatially informed histologic analysis and single-cell transcriptomic analysis that have not been described in the spatial context at single-cell resolution in human developmental liver: expression at the developing ductal plate (a layer of cells surrounding the portal tract) and CXCL12 homing. We explored these phenomena by mapping enrichment of the marker genes back to the uniform manifold approximation and projection projection and also back to the histologic samples.

Ductal plate expression is enriched for secreted phosphoprotein 1 (*SPP1*) expression (Figure 4A and B). Spatial histologic analysis at single-cell resolution (Figure 4C) and RNA single-molecule in situ hybridization (Figure 4D) highlight that the *SPP1* transcripts distinctly surround the intrahepatic veins (Figure 4C and D). Using integrated spatial transcriptomics with imaging and histologic confirmation, this finding confirms previous molecular findings of SPP1 and signal transducer and activator of transcription 1 expression in hepatobiliary hybrid progenitors at the ductal plate.^10^^,^^19^ As the *SPP1* marker was not localized on tissue in previous studies,^10^^,^^19^ this study uniquely demonstrates *SPP1* localization at the ductal plate in human developmental liver tissue using both spatial histologic analysis and in situ RNA hybridization. Our spatial findings further suggest that the expression of *SPP1* is localized to and enriched on the inner layer of the ductal plate surrounding the portal tract (Figure 4C).

Additionally, our spatial findings distinctly highlight CXCL12 expression within the single-cell analysis cluster module enrichments alongside the sinusoidal compartment that houses endothelial cells and other cell types (Figure 4A and E). Spatial histologic analysis in our samples clearly reveals *CXCL12*-expressing cells with distinct expression in and around the CD45-positive cells abutting the condensed clusters of the HSC niche. Using both spatial histologic RNA analysis (Figure 4F) and RNA single-molecule in situ hybridization (Figure 4G), these *CXCL12*-expressing cells appear within vessels and also overlap with endothelial-enriched expression in the sinusoids. The complexity of signaling factors and cellular diversity captured in this combined histologic and transcriptomic snapshot highlights the transformative potential of spatial molecular imaging not only for molecular observations at single-cell resolution but also for illuminating complex phenomena.

## Conclusion

By leveraging histologic spatial information with protein staining and transcriptomics at single-cell resolution, spatial molecular imaging allows for cell typing of functionally diverse cellular microenvironments with the capacity for capturing complex physiologic phenomena in situ at the molecular level.

The window of developmental time that we study here captures a time prior to completion of bile duct formation. The development of intrahepatic bile ducts begins when periportal hepatocytes organize into a single layer of small flat epithelial cells—the ductal plate—at 12 weeks’ gestation. By 17 to 21 weeks, areas of the ductal plate duplicate to a second layer within the nascent portal space.^20^ In neonates with complications in bile duct formation—such as biliary atresia—successful management requires timely diagnosis for optimal outcome. Spatial transcriptomics and single-molecule RNA in situ hybridization allow for histological confirmation when evaluating bile duct formation in developing human liver tissue. Continued discovery and integration of markers such as *SPP1* will enable expanded strategies in the design of molecular panels and will provide more precise evaluation of developmental timing in biliary pathobiology.

In the developing human liver, CXCL12 is produced by endothelial cells and CXC motif chemokine receptor 4 is expressed on HSCs. The concept of homing lies in the interaction between CXCL12 and CXC motif chemokine receptor 4 that guides HSCs to the liver and then to the bone marrow during development and the spatial retention of these cells due to this interaction.^21^^,^^22^ The developmental liver microenvironment influences function of the HSC niche.^9^^,^^23^ In bone marrow, the chemokine CXCL12 is responsible for the retention of hematopoietic progenitor and stem cells^24^, ^25^, ^26^ and is thought to play similar role in the developmental liver. This is supported by our spatial molecular findings localizing CXCL12 expression to within vessels and also overlapping with endothelial-enriched expression within the sinusoids.

In the future, the ability to engineer an in vitro homing mechanism and/or a multicellular environment modeled after the in situ human developmental liver environment that attracts, induces, and retains HSCs is likely on the horizon.^27^, ^28^, ^29^ The molecular spatiotemporal expression details that validate the veracity of such possible methods can be gleaned from in situ confirmation in human developmental liver tissue. Understanding the cellular interactions during this stage of liver development and hematopoiesis is crucial to recreating these cellular and signaling interactions through in vitro models.^28^ As whole-transcriptome-level single-cell spatial technologies take shape,^30^ we will see the full comprehensive picture of both new and previously described complex phenomena by molecular and histologic confirmation.

## Supplementary Materials

Supplementary Figure 3

Extended PDF
