# Supplementary figures and images for: Multimodal Spatial Transcriptomics Reveals the Developing Human Liver Niche at Single-Cell Resolution
Source: Gastro Hep Adv. 2026 Feb 3;5(4):100893. doi: 10.1016/j.gastha.2026.100893 (PMC12966723; doi:10.1016/j.gastha.2026.100893)

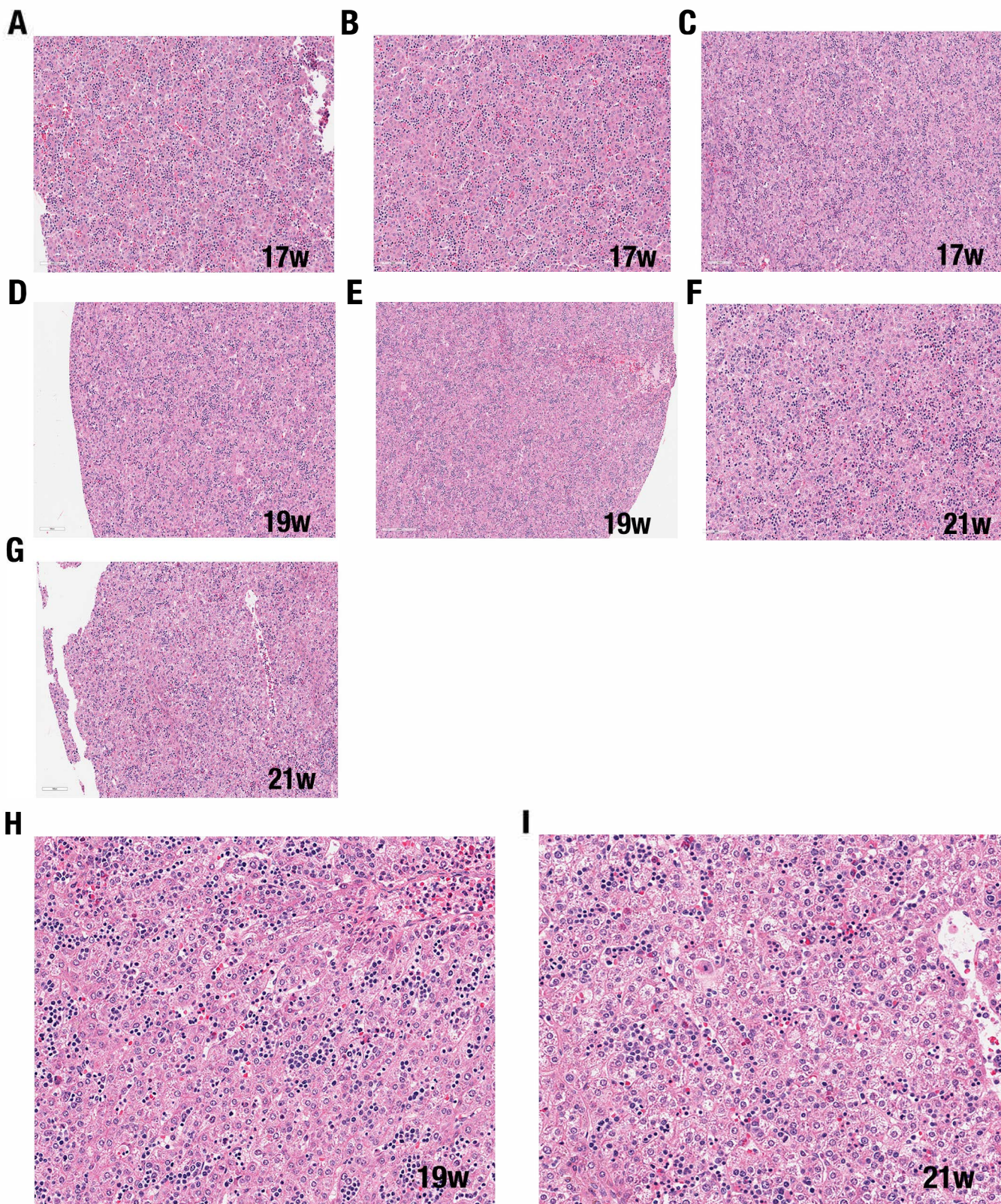

Supplement: Supplementary Figure 1 [file mmc1.pdf]

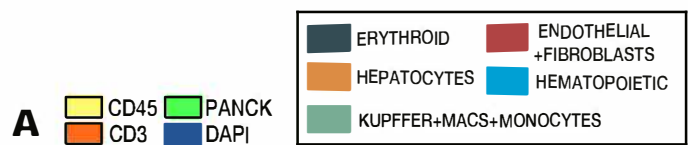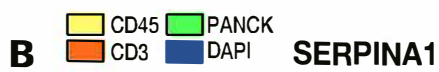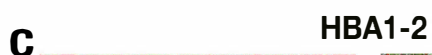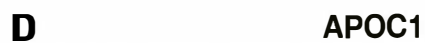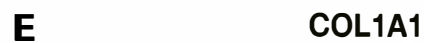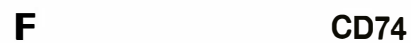

Supplement: Supplementary Figure 2 [file mmc2.pdf]

**A**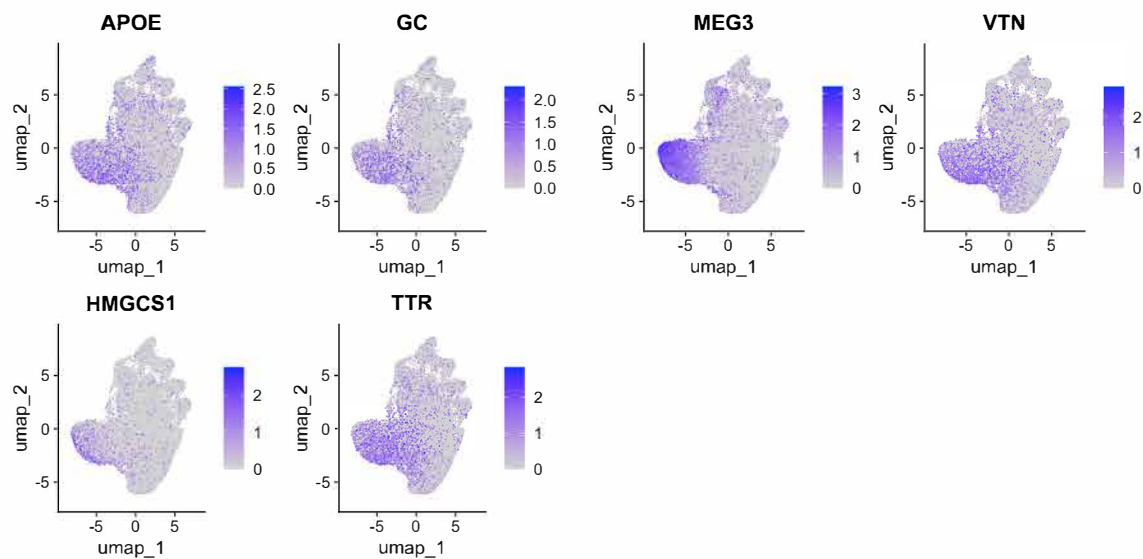**B**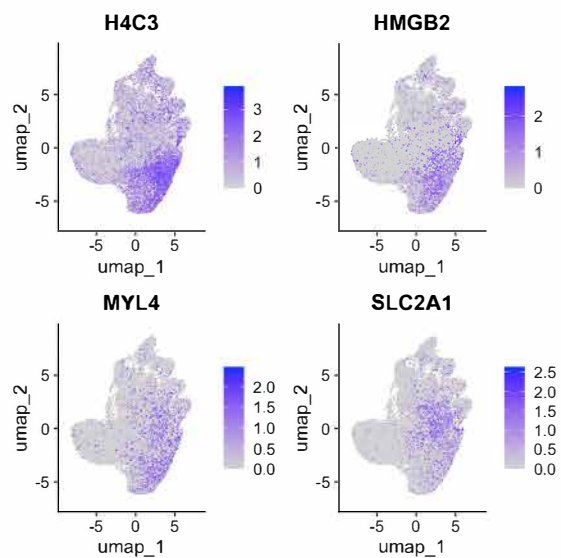

Supplement: Supplementary Figure 3 [file mmc3.pdf]
